# Timeline of cognitive impairments after radiotherapy for head and neck cancer: A review

**DOI:** 10.1016/j.ctro.2024.100890

**Published:** 2024-11-17

**Authors:** K. Wickborn, C.W.J. van der Weijden, E.F.J. de Vries, T.W.H. Meijer, M.C.A. Kramer, J.M. Spikman, A.M. Buunk, A. van der Hoorn

**Affiliations:** a Department of Radiology, University of Groningen, University Medical Center Groningen, Groningen, the Netherlands; b Department of Nuclear Medicine and Molecular Imaging, University of Groningen, University Medical Center Groningen, Groningen, the Netherlands; c Department of Radiation Oncology, University of Groningen, University Medical Center Groningen, Groningen, the Netherlands; d Department of Neurology, Unit Clinical Neuropsychology, University of Groningen, University Medical Center Groningen, Groningen, the Netherlands

**Keywords:** Head and Neck Cancer, Radiotherapy, Adverse Treatment Effects, Neurocognition

## Abstract

**Background:**

With advances in cancer treatments, long-term impairments of survivors have become more apparent. Radiotherapy of tumors in or near the brain can potentially induce cognitive impairments, impacting the quality of life of survivors. Currently, there is a lack of comprehensive information on the timeline of cognitive impairments following radiotherapy for head and neck cancer (HNC). To address this gap, we conducted a literature review on cognitive impairments observed after radiotherapy for HNC.

**Methods:**

The literature review was conducted using PubMed, Web of Science, PsycINFO, and Google Scholar. Search terms included the following keywords: head and neck tumors, radiotherapy, treatment responses, cognitive impairments, as well as variants and related subcategories.

**Result:**

Our review encompassed 23 studies involving a total of 1059 HNC patients, predominantly nasopharyngeal carcinoma. Overall, studies indicated a decline in cognitive performance post-radiotherapy compared to baseline scores, control groups, or normative data. The literature on acute effects is scarce and studies with complete neuropsychological assessments are missing. Cognitive impairments were prevalent in the majority of patients at six to 12 months post-radiotherapy, with memory deficits being the most prominent. Long-term assessments demonstrated that these cognitive deficits persisted even beyond seven years, suggesting a potentially irreversible decline in cognition following radiotherapy.

**Conclusion:**

Cognitive impairments are frequently observed at least six months after radiotherapy. Standardized cognitive assessments are imperative to evaluate impairments in individual patients. Future research in HNC should integrate neuropsychological evaluations to enhance our understanding of domain-specific impairments and the complete timeline of cognitive changes after radiotherapy.

## Highlights

• No evidence of cognitive impairments was found until 3 months post radiotherapy.
• Memory impairments can be detected as early as 3 months post radiotherapy.
• Irreversible late-delayed cognitive impairments were repeatedly found.
• Full neuropsychological assessments needed to grasp domain-specific impairments.

## Graphical abstract

**Figure:**
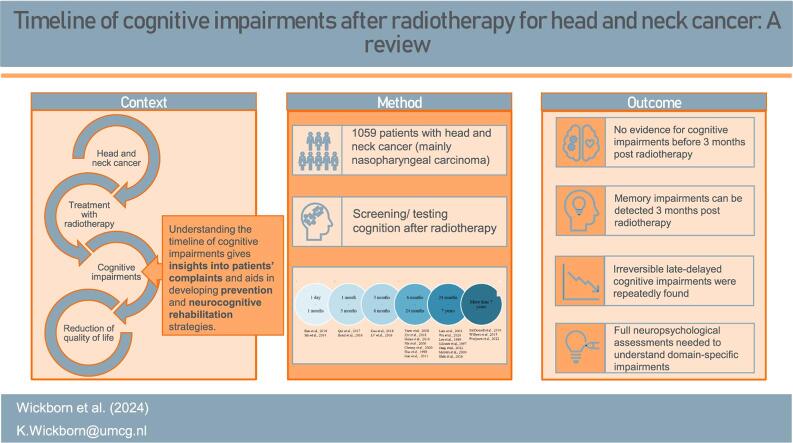

## Introduction

Radiotherapy plays an integral role in the treatment of patients with head and neck cancer (HNC). Treatment usually consists of radiotherapy with or without surgery and chemotherapy. One of the main challenges during radiotherapy is to limit the damage to surrounding tissue as much as possible. Although radiotherapeutic advances have been developed to improve target dose distribution, healthy tissue will inevitably be exposed to radiation and therefore damage to the surrounding healthy tissue cannot be prevented completely. In patients with HNC, this means that the healthy brain is often receiving some radiation. Given the location of most head and neck tumors, the brain stem, cerebellum, frontal lobes and temporal lobes (including the hippocampus) are often exposed to radiation. These structures are crucial for intact cognitive functioning, hence survivors can be at risk for adverse treatment effects [51].

Adverse effects after radiotherapy can be divided into acute effects (days to weeks after irradiation), early-delayed (one to six months after radiation), and late-delayed effects (more than six months after radiation) [47]. Most acute symptoms like pain and feeding problems due to mucositis resolve within weeks or months after irradiation. Effects on cognitive functioning, on the other hand, can be seen as delayed effects and are thought to be progressive and irreversible [13]. Common cognitive impairments can include deficits in information processing, attention, memory capacity, and executive function. These impairments, when experienced by patients with HNC, can profoundly impact their ability to engage in social, familial, and work-related activities. As a consequence, these cognitive challenges may lead to a notable decline in their overall quality of life [5], [7], [37], [48], [51].

Optimal support and management of long-term HNC survivors requires insight into common cognitive impairments, specifically into when they occur and how they progress over time. To the best of our knowledge, no review has focused on the timeline of cognitive impairments after radiotherapy yet. Therefore, the purpose of the current study was to review published articles that describe cognitive impairments after radiotherapy, focusing on the timeline of cognitive impairments after radiotherapy for HNC. With this knowledge, we hope to progress the understanding of cognitive impairments following radiotherapy in HNC survivors in order to enable early intervention and evidence-based prevention in the future.

## Methods

### Search method

A review of literature concerning cognitive impairments after radiotherapy in patients with HNC was conducted using PubMed, Web of Science, PsycINFO, and Google Scholar with no restriction on publication date or location. Search terms included the following keywords: head and neck cancer, radiotherapy, treatment side effects, cognitive impairments, as well as variants and related subcategories (full search strings can be found in the supplementary material). Additional relevant articles were identified by a backward search of reviewing the references of relevant papers, and a forward search by identifying more recent articles that cited the original paper.

### Criteria

Studies were included if they followed these main inclusion criteria: the article was published in English, the study assessed cognitive functioning with neuropsychological tests or cognitive screening (excluding self-reported cognitive complaints) of patients with HNC treated with primary or adjuvant radiotherapy, and the type of neuropsychological measurement was explicitly mentioned. There were no limitations for study sample size.

### Quality assessment

The Methodological Index for Non-Randomized Studies (MINORS; [46]) was used to assess the methodological rigor and potential risk of bias in the included studies. Each item was scored as follows: zero if not reported, one if reported but inadequate, and two if reported and adequate. The ideal total score was 16 for non-comparative studies and 24 for comparative studies (appendix). Studies were considered to have a high quality if their scoring was between 12 and 16 for non-comparative studies, and 18 to 24 for comparative studies. Studies were considered to have a moderate quality if their scoring was between eight and 11 for non-comparative studies, and 12 to 17 for comparative studies. Studies below these scores were classified as low quality (see appendix for detailed information).

### Data extraction

Studies were assessed primarily based on the title and secondly on the abstract. Then, full papers were assessed for suitability based on the above described inclusion criteria and were included if fitting. Information gathered for each study primarily concerned target population (i.e., tumor type, treatment, and patient demographics), study design, cognitive assessment, time points of assessment, and main findings.

## Results

### General characteristics and quality assessment

We identified 23 papers with a total of 1059 patients with HNC. For the comparative studies, six out of 10 studies were classified as high quality, four as moderate quality, and none were classified as low quality. For the non-comparative studies, seven out of 13 were classified as high quality, six as moderate quality and none was classified as low quality (for detailed information see appendix). Therefore, all studies were included.

Of these 23 studies, 15 studies investigated patients with nasopharyngeal carcinoma (NPC) only [11], [20], [23], [24], [27], [28], [30], [31], [32], [35], [40], [41], [45], [48], [52]. The remaining eight papers investigated a broader sample of patients with HNC [8], [17], [18], [34], [39], [50], [54], [55] including tumors of the oral cavity, larynx, hypopharynx, skull base, carcinoma of parotid, and pleiomorphic adenoma of parotid. Only nine papers specified the type of radiotherapy. Eight of these studies included patients with photon radiotherapy [23], [24], [27], [28], [35], [39], [41], [50] and one included a cohort where 80 % of the type of radiation was proton radiotherapy [18]. Details on sample characteristics and treatment per study are summarized in Table 1. Overall, nine papers assessed cognition with the Montreal Cognitive Assessment (MoCA) only. The MoCA is a ten-minute screening instrument with a total score based on aspects of executive and visuospatial functioning, naming, attention, memory, language, abstraction and orientation. Usually, a score of < 26 is considered to reflect cognitive impairment [36]. Based on the MoCA score, it is not possible to draw conclusions about impairments in specific cognitive domains.

Other common tests employed were, the Wechsler Adult Intelligence Scale (or variations), the Trail Making Task, the Stroop test, Hopkins Verbal Learning test, the Symbol digit substitution test, and the Rey Auditory Verbal Learning Test. An overview of assessed domains and tests per study can be found in Table 1. Unfortunately, the magnitude of the cognitive decline was often not reported and hence prevented conclusions about the extent of decline.

**Fig. 1:**
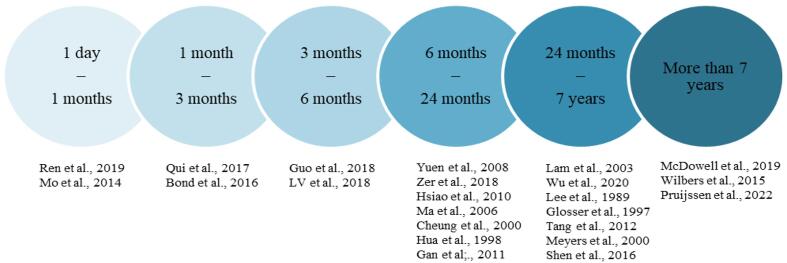
Overview of the timeframes investigated in the studies discussed in this review.

Most studies in this review considered end of radiotherapy treatment as the starting point of the post-treatment interval for cognitive assessment. For two studies, it is unclear when the interval started in relation to radiotherapy [55], [17]. For those studies it was assumed that the interval started at the end of radiotherapy treatment. Assessments performed prior to the start of treatment are here referred to as baseline assessments. Studies were sorted into the following intervals: assessment of patients one day to one months after radiotherapy, one months to three months, three months to six months, six to 24 months, 24 months to seven years, and more than seven years after radiotherapy, respectively (Fig. 1).

### Baseline assessment

From the 23 studies included, eight reported baseline assessment of cognitive functioning in patients with HNC. Of these, seven reported normal cognitive functioning at baseline. Three studies found patients with NPC to score normal on the MoCA [20], [30], [52] and one study found normal scores on the Cognitive Abilities Screening Instrument (CASI) at baseline [23]. Similarly, no difference was found between patients with NPC and healthy controls on the MoCA and Auditory verbal learning test (AVLT) [41]. One study assessed cognitive functioning in patients with chondrosarcomas of the skull base and did not find any impairments at baseline [18]. When testing a broader HNC sample, one study found that patients score similarly in cognition compared to healthy controls (with the exception of intellectual capacity, where patients performed better than healthy controls) [55].

In contrast to this, one study found a global neurocognitive impairment in 38 % of patients with HNC at baseline, whereas the highest rate of impairment was found in verbal learning [8].

### 1 day − 1 month after radiotherapy

There is limited information available on cognitive impairments in patients with HNC within the first month after radiotherapy. Only two studies in patients with NPC using mainly screening methods were reported. One study used the MoCA and AVLT to investigate cognitive functions of 20 patients one day after completing radiotherapy [41]. Post-treatment results did not significantly differ from baseline scores or from results of matched healthy controls. Similarly, another study in 51 patients showed no significant changes in scores of the Das-Naglieri cognitive assessment system (CAS) within one week after radiotherapy [35], suggesting cognitive impairment is not evident or detectable in the acute phase yet.

### 1 month – 3 months after radiotherapy

The literature regarding cognitive impairment one to three months after radiotherapy is also scarce with two studies. One study on patients with NPC (N = 39) found significantly decreased post-treatment MoCA scores compared to pre-radiotherapy results [40]. Using a more extensive neuropsychological test battery, one study found a decline in at least one cognitive domain in approximately 22 % of patients with HNC (N = 55, mainly oropharyngeal carcinoma) three months after radiotherapy. Most of these patients showing a decline in the language domain (domain-specific declines ranged from 1.8 to 12.7 %). Interestingly, 25 % of the patients improved in at least one domain over time (domain-specific improvements ranged from 0 to 7.3 %) [8].

### 3 months – 6 months after radiotherapy

Two studies investigated the MoCA scores of patients with NPC at three and six months after radiotherapy. Both studies presented findings suggesting cognitive impairments in this timeframe. A time-dependent reduction in MoCA scores was found over the timespan of three and six months post radiotherapy compared to baseline in one study (N = 27) [20]. The magnitude of these changes was not reported. The other study investigated patients with NPC at three (N = 45) and six months post radiotherapy (N = 32) and reported significant impairments of global cognitive function as measured by MoCA scores [30].

Taken together, studies that investigated adverse effects one to six months after radiotherapy for HNC are limited to four studies, of which three only included patients with NPC. In these studies, a decline in cognitive performance compared to baseline or matched healthy controls was consistently observed. However, the extent of the decline is largely unknown due to the limited information reported in most studies and the predominant use of cognitive screening methods instead of an extensive neuropsychological assessment.

### 6 months – 24 months after radiotherapy

Seven studies investigated cognition in the period between six to 24 months after treatment for HNC. One study on HNC (N = 80; mainly oropharyngeal carcinoma) assessed intellectual capacity, concentration, memory, executive function, and processing speed at baseline, and six, 12, and 24 months after [55]. Patients showed worse cognitive functioning compared to healthy controls on all timepoints. Performance decreased over the time, with 38 % of the patients showing a significant neurocognitive decline at 24 months after the initial assessment compared to their baseline scores (significant neurocognitive decline was defined as standardized regression-based score decrease of ≥ 1.64 from baseline; Cohen’s d effect size of −0.09 to −0.22). Further evidence for cognitive decline after radiotherapy was found in a study, in which patients with NPC around 18 months after radiotherapy (N = 35) were compared with matched patients with NPC before radiotherapy (N = 24). The MoCA score was significantly lower (p < 0.0001) for patients who underwent radiotherapy (mean score 24) compared to newly diagnosed patients with NPC (mean score 27) [31]. In performing a comparison to normative data, another study found that 57 % of heterogeneous patients with HNC (N = 7) scored in an impaired range on the Trail Making Test B [54]. An even higher prevalence of cognitive impairment was found in another study on patients with NPC (N = 30) around 18 months after radiotherapy [23]. Here, a significant cognitive decline compared to their baseline scores was found in 76.7 % of patients based on the Cognitive Abilities Screening Instrument scores (mean before radiotherapy: 88.9, SD = 8.19; mean after radiotherapy: 85.93, SD = 8.02; p = 0.033). Impairments were found in short-term memory, language abilities, and list-generated fluency. Evidence for impairments in language and memory was also found by another study in patients with cell carcinoma of the head and neck (N = 10; excluding nasopharyngeal tumors) on average 20 months after treatment [17]. Nine out of 10 patients showed impairments in cognitive functions across different domains of language, memory encoding and retention, attention, processing speed, and executive functioning when compared to normative neuropsychological data (70 % showed cognitive performance at least one standard deviation below normative data across cognitive domains). The most severely impaired neurocognitive functions were memory encoding (p = 0.003) and memory retention (p = 0.007). Additional deficits in specific memory areas were found in a study that assessed the neuropsychological function in patients with NPC (N = 27) with a median follow-up time of 1.7 years after radiotherapy [24]. Compared to patients with NPC awaiting radiotherapy and healthy controls, patients treated with radiotherapy showed significantly lower auditory attention and concentration, immediate and delayed verbal recall, immediate visual recall, memory and high-level visuospatial reasoning. There were no significant differences in either the visual attention test or auditory sustained attention task. Lastly, one study collected data from patients with NPC (N = 53) that finished radiotherapy at least one year before [11]. A subdivision was made between patients with NPC with and without temporal lobe necrosis (TLN). Patients with NPC without TLN performed similar to matched healthy controls. Patients with TLN showed impairment in verbal (p < 0.001) and visual memory (range, p < 0.001 to p = 0.03), language (range, p < 0.001 to p = 0.01), motor ability (p = 0.02), planning (p = 0.02), cognitive ability (p = 0.007), and abstract thinking (range, p = 0.009 to p = 0.04), whereas intelligence and attention scores were comparable to patients without TLN.

Taken together, despite differences in populations, neuropsychological measures and screening tools, the evidence from published studies suggests cognitive impairments are present at the time of six to 24 months after radiotherapy for patients with HNC (mainly NPC). Multiple studies have reported impairments in cognitive domains, such as short-term memory, language abilities, attention, processing speed, and executive function.

### 24 months – 7 years after radiotherapy

Six out of seven studies found cognitive impairments within the timeframe of two to seven years after radiotherapy. One study investigated patients with NPC with (N = 78) and without (N = 28) brain necrosis five to seven years after radiotherapy [45]. Overall, patients with radiation necrosis had lower MoCA scores (mean = 24.8), compared to patients without radiation necrosis (mean = 28.1) and healthy controls (mean = 29.1). Based on the MoCA scores, 55 % of patients with radiation necrosis showed decreased cognitive performance compared to matched healthy controls, whereas only 7 % of patients without radiation necrosis performed in the impaired range.

Similar results were found in another study in patients with NPC (N = 92), in which patients with evidence of radiation injury (N = 46) had significantly lower MoCA scores compared to those without (N = 46) after a six-year median follow-up after radiotherapy (21.32 ± 2.45 and 25.98 ± 1.73, respectively) [48]. Another study found that patients with NPC with (N = 40) and without (N = 20) evidence of temporal lobe injury (TLI) had significant lower WAIS-R scores and performed worse in information processing and comprehension scores, as well as working memory, episodic memory, and visual learning than matched healthy controls around five years after radiotherapy [27]. Of the included patients, 17 (seven without evidence of TLI, 10 with evidence of TLI) were reassessed around 28 months after the initial assessment to investigate the progress of memory function further. At this timepoint, both patient groups did not differ in verbal and visual memory tests. The only exception was that patients without evidence for TLI recalled fewer items in the 20-minute delayed recall compared to the group with evidence for TLI. In general, performances on the digit and visual span test remained stable, although there was a nonsignificant trend for improvement in verbal memory tests and a non-significant trend for worsening of visual memory [27].

Further evidence for memory impairments after radiotherapy stems from a study on patients with NPC (N = 16) with a median post-irradiation duration of 5.5 years [28]. Also here, impairments in overall cognitive functioning were found, in particular in memory aspects. More precisely, treated patients with NPC performed worse in immediate verbal and delayed nonverbal memory functioning, verbal and visual memory functioning, social comprehension, and nonverbal reasoning; and also showed a lower IQ when compared to newly diagnosed patients with NPC. In line with this, a study on patients with base of skull tumors of the paranasal sinuses (N = 19) found memory impairments in 80 % of treated patients after a mean follow-up of 73 months after radiotherapy, when compared to normative data [34]. More specifically, verbal memory was the most significantly affected domain, with more than half of the patients having difficulty learning new information and 80 % having accelerated forgetting of the information over time. One third of the sample had difficulty with visual-motor speed, executive functions, and fine motor coordination. The majority of patients had normal or close to normal intellectual functions and attentional abilities. In another study on patients with NPC (N = 54) MoCA scores reduced over time, with 35 % of patients experiencing cognitive impairments two years after radiotherapy and 16 % of patients having a decline of three or more in the MoCA scores [52].

In contrast to most studies, one study did not find evidence for adverse cognitive changes after radiotherapy for chordomas and low grade chondrosarcomas of the base of skull (N = 17). No decline in general intelligence, language, memory, and higher-level attentional functions was found at a mean follow-up of 25 and 47 months after radiotherapy, when compared to baseline [18].

Taken together, six out of seven papers included here investigated patients with NPC who undergo radiotherapy within the timeframe two to seven years before cognitive assessment. The majority of these papers found evidence for cognitive impairments after radiotherapy. Especially, patients with radiation necrosis or patients with evidence of TLI may have a higher risk for developing cognitive impairments. The only study that investigated changes in cognition in the population of chordomas and low grade chondrosarcomas of the base of skull did not find evidence of adverse cognitive changes after radiotherapy.

### More than 7 years after radiotherapy

Based on three studies, patients with HNC seem to experience cognitive impairments seven and more years after radiotherapy. One study that included patients with NPC (N = 102) found that 70 % had a MoCA score below 26; and 32 % had a score below 23 after a median 7.5 year follow-up [32]. Based on more extensive neuropsychological assessments, around 17 % of benign or malignant HNC tumor patients (N = 29, mainly lymphoma, pleiomorphic adenoma of parotid, and carcinoma of parotid) with a median post-radiotherapy time of 9.2 years showed worse episodic memory but no significant difference in working memory, executive functioning, verbal fluency, or speed of information processing [39]. Another study conducted on 44 HNC patients (mainly larynx) found that approximately 11 % of patients had impaired episodic memory, and around 7 % had impaired information processing speed after a mean follow-up of 6.7 years after radiotherapy. However, no significant differences were found in working memory, executive function and verbal fluency [50]. In conclusion, patients with HNC treated with radiotherapy appear to experience cognitive impairments after seven and more years. Up to 70 % of patients scored in an impaired range of MoCA scores, with deficits in episodic memory being experienced most.

## Discussion

This study aimed to review the literature on cognitive impairments in HNC patients after radiotherapy, ranging from acute effects to effects found years after treatment. Unfortunately, only two studies investigated the acute effects of radiotherapy on cognitive functioning. According to these studies, acute cognitive effects may not be immediately evident after radiotherapy. However, only cognitive screening methods were performed and solely patients with NPC were included. Further research, encompassing diverse HNC populations and comprehensive neuropsychological assessments, is required to confirm the absence of acute cognitive effects post-radiotherapy.

The literature on effects of radiotherapy assessed one to six months after irradiation remains scarce, with only four available studies. All studies found evidence of adverse-effects on cognition. As three studies relied solely on the MoCA, it is not possible to draw conclusions about domain-specific cognitive impairments. Also here, these studies focused on patients with NPC. Further research with diverse HNC samples and more extensive neuropsychological assessments is needed to fill current knowledge gaps.

More studies assessed effects of radiotherapy after six months. Here, the vast majority of papers show that a significant percentage of patients with NPC suffer from neurocognitive deficits after radiotherapy. Overall, the timeline of the included studies suggests that cognitive side effects of radiotherapy are not present or detectable with a general screening test in the acute phase yet, but start to become overt one month after the completion of radiotherapy [10], [52]. Due to the scarcity of studies and used screening tests investigating the earlier effects after radiotherapy, it is not possible to draw conclusions about which domains are affected during this early phase and when they occur. However, it is plausible that subclinical effects, which lead to the cognitive impairments that are observed one month after radiotherapy, are already present. It is therefore highly relevant to identify these subclinical processes, as they may be used as predictors for delayed adverse events.

According to the published studies that assessed adverse effects six months after radiotherapy, impairments in episodic memory are the most consistent finding. Results regarding impairments of attention, language, and executive functioning after radiotherapy are inconsistent between studies. This might be explained by differences in patient characteristics, such as the location and size of the primary tumor and therefore differences in treatment modalities, including radiation dose to the brain. These factors can have an impact on the nature and extent of adverse neurocognitive effects.

HNC is a heterogeneous disease, including tumors of for example the oral cavity, nasopharynx, larynx, and salivary glands, with grossly varying patient characteristics and survival rates. Most of the included studies (N = 15) investigated impairments in patients with NPC only. For NPC, areas like the brain stem, cranial nerves, and parts of the inferomedial temporal lobe and hippocampus are often inevitably exposed to radiation. The hippocampus plays a crucial role in learning and memory, with adult neurogenesis primarily occurring in its dentate gyrus and subgranular zone [26], as well as the subventricular zone of the lateral ventricles [15]. Studies in animal models suggest that radiation exposure can impair neurogenesis in these areas, inhibiting the differentiation of neural progenitor cells into mature neurons [43], [42]. Clinical studies suggest these findings apply to humans as well [49]. For example, hippocampal doses exceeding 7.3 Gy in patients with benign or low-grade brain tumors were associated with long-term cognitive impairment, leading to the development of hippocampus-sparing strategies [19].

Besides the hippocampus, radiotherapy-induced injuries to the temporal lobe are reported to be a potential neurological complication following radiotherapy in NPC [11], [12]. TLI has been linked to impaired development in memory and learning [1], [4], [11], [44]. Multiple studies investigated the impact of TLI/TLN or radiation dose to the temporal lobe on cognitive effects. One of the studies concluded that TLN is a contributing factor for develop cognitive impairments [11]. Interestingly, in this study no significant difference in total tumor dosage and dosage per fraction was found between patients who developed TLN and patients who did not. In another study, patients that received a mean dose to the temporal lobes greater than 36 Gy were found to decline more in cognitive function compared to those that received less than 36 Gy. Similarly, patients for whom the percentage of temporal lobe volume that received > 60 Gy was greater than 10 % showed a more pronounced cognitive decline than those with equal or smaller than 10 % [23]. Other studies also found that radiation dose to the temporal lobe was moderately linked to the time to complete the Trail Making Test-B [54] and worse performance on memory decoding [17]. Contrary to the aforementioned studies, one study did not find significant differences in cognitive performance between patients with and without evidence of TLI [27]; and another study found that the MoCA scores did not correlate with TLN [32]. Taken together, evidence from the majority of studies hints to a link between irradiation of the temporal lobes and the extent of cognitive impairments after radiotherapy, however, the link might be more complex than radiation dose to the temporal lobes alone. With advancements in radiotherapy treatment-planning techniques, it is possible to spare healthy tissues and specific structures that are potentially more sensitive to radiation. Intensity-modulated radiotherapy (IMRT) and Volumetric Modulated Arc Therapy (VMAT) offer a more precise dose delivery due to three-dimensional control [23], thereby decreasing dose to normal tissues [25], [38]. However, the link between low dose bath to structures of the central nervous system and resulting neurocognitive outcomes is unclear. Here, nine out of 11 papers still found neurocognitive decline in patients with HNC (vast majority NPC) treated with IMRT [17], [20], [23], [30], [31], [32], [40], [45], [52], in contrast to [41] and [35]. Nowadays, proton therapy is used more and more to irradiate HNC tumors. Protons progressively lose energy while interacting with surrounding materials along their path until they come to a complete stop. The dose deposited along the beam path remains relatively constant until reaching the end of the range, creating a distinctive Bragg peak where the dose peaks and then rapidly diminishes to near-zero. With this, proton therapy aims to reduce damage to surrounding tissue, and has consequently the potential to reduce adverse effects on cognition. In this review, only one study investigated adverse effects in chordomas and low-grade chondrosarcomas of the base of skull that were mainly treated with proton radiotherapy [18]. It is not possible to compare the magnitude of cognitive decline between different radiotherapy strategies due to missing data and the differences in patient groups, cognitive tests and additional treatments between studies [33].

Based on the studies included in the present review, the influence of radiotherapy dosage on cognitive impairments directly is inconclusive in patients with HNC. Some of the studies included in this review found a relationship between overall received dose to the tumor and cognitive impairments (i.e., [34] and [52]), or mean doses to the temporal lobes and cognitive impairment (i.e., [17] and [23]), while others did not find a significant link (i.e., [27] and [32]). Still many knowledge gaps remain and the link between received doses, radiotherapy-sensitive brain areas and domain-specific cognitive impairments should be further investigated in well-designed prospective clinical trials.

From the studies included in this review, 39 % (N = 9) assessed cognition by the MoCA. Although the MoCA is an easy and accessible test, it remains a brief and rapid screening test that does not allow to conclude about deficits in specific cognitive domains and can be insensitive to subtle changes in cognition. Moreover, future studies should be aware that correcting cut-off scores for age, education, race and ethnicity is paramount in order to ensure sufficient specificity, sensitivity, and predictive value of the MoCA. To gain more insights into domain-specific cognitive changes over time, future research should not only employ the MoCA, but also extensive neuropsychological assessments performed by clinical psychologists. More sensitive neuropsychological test would enable more accurate assessment whether subtle cognitive impairments in patients already existed before treatment, increase in severity over time, or only start to occur months or years after treatment. Longitudinal studies with a sensitive test battery could thus provide insights into the true onset of cognitive impairments, and allow for a clearer understanding of how they evolve over time.

It is noteworthy to say that despite the evidence for cognitive impairments after radiotherapy, it is impossible to quantify the radiation-induced cognitive impairments in isolation of other treatment modalities. Patients with HNC can receive surgery and chemotherapy in addition to radiotherapy. None of the studies included in this review addressed the potential confounding effects of surgery on cognitive outcomes. Only four studies included here investigated patients treated with radiotherapy only. From these studies, three found a decrease in cognitive performance after radiotherapy (i.e., [24], [27], [28]; in contrast to [18]). Five studies investigated the effects of chemotherapy in addition to radiotherapy, and four of them did not find a link between chemotherapy and cognitive outcomes (i.e., [17], [23], [32], [34]; in contrast to [48]), suggesting the effect of radiotherapy on cognitive decline is more pronounced than that of chemotherapy. This is in contrast to multiple other studies that found cognitive impairments after chemotherapy only in different cancer groups and hence coining the term “chemobrain” [14], [22]. However, little is known about chemotherapy-induced cognitive impairments in patients with HNC. Further research on both, chemotherapy-induced cognitive impairments and radiotherapy-induced cognitive impairments in patients with HNC is warranted to quantify the effects of these treatments in isolation.

Developing HNC and experiencing cognitive impairments share multiple risk factors. Tobacco is a risk factor for HNC [6], [21], [29], [53] as well as for neurocognitive impairments [3], [9]. Similarly, alcohol consumption is a risk factor for HNC [6], [21], [29] and for neurocognitive impairments [2], [16]. As a result, patients might already experience cognitive impairments before radiotherapy. A study in 209 patients with HNC found that 67.7 % of patients already scored in an impaired cognitive range before treatment (cohort mean MoCA score of 23.01; [51]). However, the vast majority of studies that reported baseline scores, did not find a difference in general intelligence or cognitive performance in patients at baseline (i.e., [18], [20], [23], [30], [41], [52], [55]; in contrast to [8]). It is nevertheless recommended to compare the cognitive data to an individual baseline assessment for each participant. This is both to monitor changes over time, and also to compensate for baseline cognitive impairments that can be caused by overall poor health, smoking, alcohol consumption and comorbidities. Cognitive impairments at baseline or in the trajectory of treatment could translate to difficulties in understanding the treatment plan that is often complex and multi-modal. Clinicians, researchers and patients should be aware of the risk of cognitive impairments and adjust the frequency and extent of follow-ups, in addition to offering support during and after treatment.

## Conclusion

The vast majority of studies that investigate cognitive impairment in patients with NPC after radiotherapy show adverse effects after one month in a significant number of patients, with literature suggesting similar findings in other HNC populations. However, studies on acute effects after radiotherapy are almost entirely missing. Additionally, cognitive impairments could be tumor site-specific and the link between received doses, radiotherapy-sensitive brain areas, and domain-specific cognitive impairments should be further investigated in different patient populations. Longitudinal studies that use extensive neurocognitive test batteries at baseline and follow-up, and that also report the magnitude of decline, are scarce. Detailed information from these neurocognitive assessments is crucial to increase our understanding of domain-specific impairments, which may enable the unravelling of the full timeline of cognitive impairments in patients with HNC after radiotherapy. With this knowledge, further steps into the development of evidence-based prevention, early intervention, and neurocognitive rehabilitation can be taken.

## Funding

Prof. van der Hoorn received funding of a personal NWO VENI grant (VENI 09150161910041).

## Declaration of Competing Interest

The authors declare the following financial interests/personal relationships which may be considered as potential competing interests: [Prof. Dr. E.F.J. de Vries declares financial support from Hoffmann-La Roche, Eli Lilly, Bristol Myers Squibb, Ionis Pharmaceutical, Rodin Therapeutics, Lysosomal Therapeutics, Novartis, Janssen-Cilag BV, GE Healthcare and GlaxoSmithKline, for contracted research not related to this study, paid to the institution in the past 5 years.

Prof. van der Hoorn is a Medical Annotation specialist for Quantitas Solutions. If there are other authors, they declare that they have no known competing financial interests or personal relationships that could have appeared to influence the work reported in this paper].

## Figures and Tables

**Table:** 

(((((“head and neck neoplasms”[MeSH Terms] OR (“head”[All Fields] AND “neck”[All Fields] AND “neoplasms”[All Fields]) OR “head and neck neoplasms”[All Fields] OR (“head”[All Fields] AND “neck”[All Fields] AND “cancer”[All Fields]) OR “head and neck cancer”[All Fields] OR (“oropharyngeal neoplasms”[MeSH Terms] OR (“oropharyngeal”[All Fields] AND “neoplasms”[All Fields]) OR “oropharyngeal neoplasms”[All Fields] OR (“oropharyngeal”[All Fields] AND “carcinoma”[All Fields]) OR “oropharyngeal carcinoma”[All Fields]) OR (“nasopharyngeal carcinoma”[MeSH Terms] OR (“nasopharyngeal”[All Fields] AND “carcinoma”[All Fields]) OR “nasopharyngeal carcinoma”[All Fields]) OR ((“hypopharynx”[MeSH Terms] OR “hypopharynx”[All Fields] OR “hypopharyngeal”[All Fields]) AND (“carcinoma”[MeSH Terms] OR “carcinoma”[All Fields] OR “carcinomas”[All Fields] OR “carcinoma s”[All Fields])) OR (“salivary gland neoplasms”[MeSH Terms] OR (“salivary”[All Fields] AND “gland”[All Fields] AND “neoplasms”[All Fields]) OR “salivary gland neoplasms”[All Fields] OR (“salivary”[All Fields] AND “gland”[All Fields] AND “cancer”[All Fields]) OR “salivary gland cancer”[All Fields]) OR (“tongue neoplasms”[MeSH Terms] OR (“tongue”[All Fields] AND “neoplasms”[All Fields]) OR “tongue neoplasms”[All Fields] OR (“tongue”[All Fields] AND “cancer”[All Fields]) OR “tongue cancer”[All Fields]) OR (“mouth neoplasms”[MeSH Terms] OR (“mouth”[All Fields] AND “neoplasms”[All Fields]) OR “mouth neoplasms”[All Fields] OR (“oral”[All Fields] AND “cavity”[All Fields] AND “cancer”[All Fields]) OR “oral cavity cancer”[All Fields]) OR ((“paranasal sinuses”[MeSH Terms] OR (“paranasal”[All Fields] AND “sinuses”[All Fields]) OR “paranasal sinuses”[All Fields]) AND (“cancer s”[All Fields] OR “cancerated”[All Fields] OR “canceration”[All Fields] OR “cancerization”[All Fields] OR “cancerized”[All Fields] OR “cancerous”[All Fields] OR “neoplasms”[MeSH Terms] OR “neoplasms”[All Fields] OR “cancer”[All Fields] OR “cancers”[All Fields]))) AND (“neurocognition”[All Fields] OR “neurocognitive”[All Fields] OR “neurocognitively”[All Fields])) OR (“cognition”[MeSH Terms] OR “cognition”[All Fields] OR “cognitions”[All Fields] OR “cognitive”[All Fields] OR “cognitively”[All Fields] OR “cognitives”[All Fields]) OR (“memories”[All Fields] OR “memory”[MeSH Terms] OR “memory”[All Fields] OR “memory s”[All Fields]) OR ((“intelligence”[MeSH Terms] OR “intelligence”[All Fields] OR “intelligences”[All Fields] OR “intelligent”[All Fields] OR “intelligently”[All Fields] OR “intelligibilities”[All Fields] OR “intelligibility”[All Fields] OR “intelligible”[All Fields]) AND (“quotient”[All Fields] OR “quotients”[All Fields])) OR (“attention”[MeSH Terms] OR “attention”[All Fields] OR “attentions”[All Fields] OR “attention s”[All Fields] OR “attentional”[All Fields] OR “attentive”[All Fields] OR “attentively”[All Fields] OR “attentiveness”[All Fields])) AND (“cognition”[MeSH Terms] OR “cognition”[All Fields] OR “cognitions”[All Fields] OR “cognitive”[All Fields] OR “cognitively”[All Fields] OR “cognitives”[All Fields])) OR (“language”[MeSH Terms] OR “language”[All Fields] OR “languages”[All Fields] OR “language s”[All Fields] OR “programming languages”[MeSH Terms] OR (“programming”[All Fields] AND “languages”[All Fields]) OR “programming languages”[All Fields]) OR (“executive function”[MeSH Terms] OR (“executive”[All Fields] AND “function”[All Fields]) OR “executive function”[All Fields])) AND (“radiotherapy”[MeSH Terms] OR “radiotherapy”[All Fields] OR “radiotherapies”[All Fields] OR “radiotherapy”[MeSH Subheading] OR “radiotherapy s”[All Fields])

**Table 1:** Individual minors score.

**Study**	Clearly stated aim	Inclusion of consecutive patients	Prospective data collection	Endpoints appropriate to study aim	Unbiased assessment of study endpoint	Follow-up period appropriate to study aim	<5% lost to follow-up	Prospective calculation of study size	Adequate control group	Contemporary groups	Baseline equivalence of groups	Adequate statistical analyses	Total	**Quality assessment**
**Ren**	2	1	2	2	1	2	2	0	2	2	2	2	20/24	**High**
**Mo**	2	2	2	2	2	1	1	0	NA	NA	NA	NA	12/16	**High**
**Qiu**	2	1	2	2	1	2	1	0	NA	NA	NA	NA	11/16	**Moderate**
**Bond**	2	2	2	2	1	2	1	0	NA	NA	NA	NA	12/16	**High**
**Guo**	2	1	2	1	1	2	1	0	1	1	1	2	15/24	**Moderate**
**Lv**	2	1	2	2	2	1	1	0	1	2	1	1	16/24	**Moderate**
**Yuen**	1	1	2	1	1	NA	NA	0	NA	NA	NA	NA	6/12	**Moderate**
**Zer**	2	2	2	1	2	2	2	0	NA	NA	NA	NA	12/16	**High**
**Hsiao**	2	1	2	2	2	2	2	0	NA	NA	NA	NA	13/16	**High**
**Ma**	2	1	2	1	1	NA	NA	0	2	1	1	2	13/20	**Moderate**
**Cheung**	2	1	2	2	2	NA	NA	0	2	2	2	2	17/20	**High**
**Hua**	2	1	2	1	2	NA	NA	0	2	2	2	2	16/20	**High**
**Gan**	2	2	2	1	1	NA	NA	0	NA	NA	NA	NA	8/12	**Moderate**
**Lam**	2	1	2	1	1	NA	NA	0	2	2	1	1	13/20	**Moderate**
**Wu**	2	2	2	2	1	1	1	0	NA	NA	NA	NA	11/16	**Moderate**
**Lee**	1	2	2	2	1	NA	NA	0	2	2	2	2	16/20	**High**
**Glosser**	2	1	2	2	1	1	2	0	NA	NA	NA	NA	11/16	**Moderate**
**Tang**	2	2	2	2	1	NA	NA	0	2	2	1	2	16/20	**High**
**Meyers**	2	2	2	1	2	NA	NA	0	NA	NA	NA	NA	9/12	**High**
**Shen**	2	2	2	2	1	2	2	0	1	2	1	1	18/24	**High**
**Wilbers**	2	1	2	1	2	NA	NA	0	NA	NA	NA	NA	8/12	**Moderate**
**McDowell**	2	2	2	2	2	NA	NA	0	NA	NA	NA	NA	10/12	**High**
**Pruijssen**	2	2	2	1	2	NA	NA	0	NA	NA	NA	NA	9 /12	**High**

## Appendix A

**Data source and keywords:**

Search string for Pubmed, EMBASE, Web of ScienceSearch string google scholar

Head and neck cancer OR Head and neck OR head and neck tumors AND cognitive impairments OR adverse effects OR treatment response OR cognitive decline AND radiotherapy OR irradiation OR radiation

Explanation of items (from [46])

Items are scored as 0 (not reported), 1 (reported but inadequate), or 2 (reported and adequate).

Clearly Stated Aim: The research question should be precise and relevant based on the existing literature.

Inclusion of Consecutive Patients: All patients who meet the inclusion criteria should be included in the study during the designated study period.

Prospective Data Collection: Data should be collected following a protocol established prior to the start of the study.

Endpoints Appropriate to the Study Aim: The criteria used to evaluate the primary outcome should be clearly explained and aligned with the study’s objectives. The endpoints should be assessed on an intention-to-treat basis.

Unbiased Assessment of the Study Endpoint: Objective endpoints should be evaluated blindly, and subjective endpoints should be double-blind evaluated. If blinding is not done, the reasons should be clearly stated.

Follow-up Period Appropriate to the Study Aim: The follow-up duration should be long enough to assess the primary outcome and any potential adverse events.

Loss to Follow-Up Less Than 5 %: All patients should be included in the follow-up. If not, the proportion lost to follow-up should not exceed the proportion experiencing the primary endpoint.

Prospective Calculation of Study Size: The study should include information about the size of the detectable difference of interest, with a 95 % confidence interval calculation based on the expected incidence of the outcome. It should also include the level for statistical significance and estimates of power when comparing outcomes.

Additional Criteria for Comparative Studies:

Adequate Control Group: The control group should have a gold standard diagnostic test or therapeutic intervention, recognized as the optimal approach based on available published data.

Contemporary Groups: The control and study groups should be managed within the same time period (no historical controls).

Baseline Equivalence of Groups: The groups should be comparable in terms of criteria other than the studied endpoints. There should be an absence of confounding factors that could bias the interpretation of results.

Adequate Statistical Analyses: The statistical methods should be appropriate for the type of study, including the calculation of confidence intervals or relative risk.

Some studies did only assess one timepoint, therefore the criteria regarding follow-up were not applicable. Calculations were made based on possible scores and where adjusted if necessary.

**For non-comparative studies with a maximum of 16:**

High Quality: Scores of 12 to 16 (100 % – 75 %).

Moderate Quality: Scores of 8 to 11 (50 % − 75 %).

Low Quality: Scores below 8 (less than 50 %).

Adjusted scores

**For Non-Comparative studies with maximum score of 12:**

High Quality: Scores of 9 to 12.

Moderate Quality: Scores of 6 to 8.

Low Quality: Scores below 6.

**For Comparative studies with a maximum of 24:**

High Quality: Scores of 18 to 24 (100 % – 75 %).

Moderate Quality: Scores of 12 to 17 (50 % − 75 %).

Low Quality: Scores below 12 (less than 50 %).

## Adjusted scores

**For Comparative Studies with maximum score of 20:**

High Quality: Scores of 15 to 20.

Moderate Quality: Scores of 10 to 14.

Low Quality: Scores below 10.
