# Supplementary material for: Timeline of cognitive impairments after radiotherapy for head and neck cancer: A review
Source: Clin Transl Radiat Oncol. 2024 Nov 17;52:100890. doi: 10.1016/j.ctro.2024.100890 (PMC11847131; doi:10.1016/j.ctro.2024.100890)
Supplement: Supplementary data 1 [file mmc1.docx]

| Reference  year | Study type | N | Tumor type | Tx intent  Tx form  (Dose) | Age in yr  Mean\|Median (SD\|Range) | % male | Timepoints  Mean\|Median  (Range) | Cognitive Domains:  Tests / Subtest | Comparison  (N) | Findings  (pre-RT± SD/post-RT ± SD) |
| --- | --- | --- | --- | --- | --- | --- | --- | --- | --- | --- |
| Ren  2019 | Pros | 20 | NPC | Primary  IMRT  Photon  (70–80 Gy; 33-36 fr.)  60% CT | Mean= 46.30  (8.1) | 80 | 1 d. pre RT  1 d. post RT | Screening neurocognitive function:  MoCA  Learning & memory: AVLT | Within patient  Matched HC (17) | MoCA mean: 27.25 ± 1.68/27.00 ± 2.05  AVLT mean:  Immediate recall: 9.97 ± 2.03/10.42 ± 2.35  Delayed recall: 10.30 ± 3.23/10.80 ± 3.25  Delayed recognition: 13.95 ± 1.32/13.55 ± 1.43  No sig. diff. pre & post RT  No sig. diff. to HC |
| Mo  2014 | Pros | 51 | NPC | Primary  IMRT  Photon  (70–72.32 Gy)  Maj. 2-3 cycles CT | Mean= 40.1 (8.7) | 63 | Pre RT  <1 w. post RT | Planning, attention, simultaneous & successive processing:  DN: CAS | Within patient | No sig. diff. pre & post  BL scores n.r. |
| Qiu  2018 | Pros | 39 | NPC | Primary  IMRT  (68-70 Gy; 30-33 fr.; 2.2-2.27 Gy/fr.) | Mean= 40,5  (8.4) | 64 | Pre tx  3 mo. post RT | Screening neurocognitive function:  MoCA | Within patient | Sig. reduced MoCA 3 mo. Post tx (p < 0.05)  Magnitude n.r.  BL score n.r. |
| Bond  2016 | Pros | 55 | HNC  30 OPC  5 NPC  1 HP  5 Lx  6 PNS/NC  1 SG  4 UP  3 Or | Primary/adjuvant  CR  (n.r.)  (n.r.) | Mean= 55.1 (33- 70) | 78 | Pre Tx  3 mo. Post RT  (2.4-5.9 mo) | Attention/ processing speed:  TMT-A  Executive function:  TMT-B  Language:  AVF  Verbal memory:  RAVLT total  RAVLT delayed recall  Processing speed:  SDMT  Screening neurocognitive function:  MMSE | Within patient | 38 % global neurocognitive impairment at BL  54.5% no sig. diff. pre & post  21.8 % decline in at least 1 domain  (13% in language; domain-specific declines from 1.8- 12.7%)  25.4% improved in at least one domain  (in attention/concentration, executive function, verbal learning; domain-specific improvements from 0-7.3%)  MMSE n.r. |
| Guo  2018 | Pros | Pre RT: 63  3 mo: 38  6 mo: 27 | NPC | Primary  IMRT  (68–70 Gy; 30–33 fr.;  2.12–2.33 Gy/fr.)  93.7% CR | Mean= 39.7 (9.3) | 72 | Pre RT 3 mo post RT 6 mo post RT | Screening neurocognitive function:  MoCA | Within patient  HC (20) | No sign. Diff. to HC at BL  Time-dependent lower MoCA scores post tx when compared with BL  (Magnitude n.r.) |
| Lv  2018 | Pros | Pre-RT: 50  3 mo: 32 6 mo: 23 | NPC | Primary  IMRT or tRT  (68 – 70 Gy; 30-33 fr.; 2.12-2.33 gy/ fr.) | Mean= 39.9 (9.58)  (21-62) | 67 | Pre tx  3 mo post RT  6 mo post RT | Screening neurocognitive function:  MoCA | Pros  Matched HC  (20) | No sign. Diff. to HC at BL  “Cognitive function was sig. impaired” after  (Magnitude n.r.) |
| Yuen  2008 | Pros | 7 | HNC  1 N SSC  1 T&E SSC  1 PDC  1 NPC  1 T SSC  1 DLBncHl  1 Ton SSC | Primary  RT, CR  (max. 70Gy; 35 fr.,) | Mean= 57.6  (8.8) | 86 | 2 within RT  5 post RT  (1 w.; 1.4 mo.; 4.9 mo.; 24.3 mo.; 32.3 mo.) | Executive function:  TMT-B | Normative data | TMT-B median score: 92s  57% impaired cognition  43% more than 180s or unable to complete test  Negative correlation between TMT-B & duration post RT (maybe resolve over time)  Intelligence scores n.r. |
| Zer  2018 | Pros | 80 | n.mo. SCC HNCs  excl. NPC  5 HPX  61 OPC  7 Lx  2 NC  5 UP | Primary  RT, CR  (max. 70Gy; 35 fr. 71) | Mean= 58.3 (7.6) | 85 | Pre tx  6 mo. Post BL  12 mo. Post BL 24 mo. Post BL | Intellectual capacity:  WAIS-III Vocabulary  WAIS-III Matrix Reasoning  Attention/concentration:  WMS-III Digit span forward  WMS-III Spatial span forward  Visual Memory:  BVMT forms 1 & 3 (Total Recall; Delayed Recall)  Verbal memory:  HVLT-R forms 1 & 5 (Total Recall; Delayed Recall)  Attention/processing speed:  DKEFS Color naming  DKEFS Word reading  TMT-A  Executive function:  WFT  DKEFS Stroop Inhibition  TMT-B  WMS-III Digit Span Backward  WMS-III Spatial Span Backward  Manual dexterity:  PPB  Global cognitive function composite:  Mean score across domains | Within patient  HC (40) | BL: pts & HC scores similar, except intellectual capacity, where pts performed better  pts deficits increased over time, including intellectual capacity, concentration/short-term attention span, verbal memory, executive functioning, global cognitive function composite  Pts did not decline immediately, but in time post tx  38% of pts showed neurocognitive decline at 24 mo.  Cohen d, ES [95%Cis] for 6, 12, & 24 mo post RT:  Intellectual capacity: −0.46 [−0.64 to 0.30], −0.51 [−0.72 to −0.30], & −0.70 [−0.92 to −0.49]  concentration/short-term attention span: −0.19 [−0.37 to 0.00], −0.38 [−0.55 to −0.21], −0.54 [−0.71 to −0.37]  verbal memory: −0.16 [−0.33 to 0.02], −0.38 [−0.64 to −0.12], −0.53 [−0.74 to −0.32]  executive function: −0.14 [−0.27 to 0.00], −0.34 [−0.52 to −0.16], −0.43 [−0.64 to −0.22]  global cognitive function composite: −0.38 [−0.55 to −0.22], −0.75 [−0.92 to −0.58], −1.06 [−1.26 to −0.86] |
| Hsiao  2010 | Pros | 30 | NPC | Primary  IMRT  Photon  (70-72Gy; 1.8-2Gy fr.)  87% CT | Mean= 47  (11.6) | 77 | Pre RT 12- 26 mo. Post RT  (mean 18 mo.) | Screening neurocognitive function:  CASI | Within patient | decreased cognitive functioning scores in 23 pts (76,7%) post-Rt compared to pre-RT (Mean pre RT: 88.9 (SD=8.19); Mean post RT: 85.93 (SD=8.02))  Areas involved are short term memory, language abilities, list-generating fluency  Pts with mean dose to t.l.s of greater than 36 Gy decline more compared to those that received less than 36 Gy (p = 0.017)  Pts for whom % of t.l. volume that received >60 Gy was >10% showed higher cognitive decline than those ≤10%  no associations between cognitive functioning decline & CT, s, age, gender, education, hypertension, diabetes, smoking history  At BL pts had normal scores  No TLN |
| Ma  2016 | Pros | 35 | NPC | Primary  IMRT  (58 – 70Gy:30–33 fr.)  CT | Mean= 42.09  (8.44)  (20 – 55) | 80 | 6–87 mo. Post RT  (mean 18 mo.) | Screening neurocognitive function:  MoCA | Matched untreated NPC (24) | NPC pts with RT had sig. lower MoCA scores compared to newly diagnosed NPC  (MoCA score of 27 vs, 24, P < 2.70e – 13) |
| Cheung  2000 | Pros | 53 | NPC  22 TLN-  31 TLN+ | Primary  RT alone  (TLN-:58,1 (+/- 4.5), 2.8 (+/- .7) Gy/fr.)  (TLN+: 58,4 (+/- 4.5), 2.7 (+/- .7)Gy/ fr.) | TLN-:  Mean= 52  (10.9)  TLN+:  Mean= 57  (9.4) | TLN-: 59  TLN+: 80 | >1 yr. post RT  Mean/median n.r. | IQ score& verbal performance:  WAIS-R  Screening neurocognitive function:  MMSE  Attention:  CTT (Part1)  WAIS-R (Digit symbol & Digit span)  Memory:  HKLLT  WMS-R (logical memory)  Visual memory:  BVMT-R  WMS-R (visual reproduction)  Expressive language:  VFT  BNT (short form)  Verbal comprehension:  WAIS-R (information & comprehension)  Visual ability:  WMS-R (visual reproduction)  WAIS-R (block design)  Facial recognition ability:  FRT  Manual dexterity:  PPB  Planning & organization:  RFT (copy trial)  Verbal concept formation:  WAIS-R (similarities subtest)  Concept thinking test  Common knowledge test  Cognitive flexibility:  CTT (Part 2)  Perseveration:  FPT | Matched  HC (31)  TLN+ (22)  TLN- (31) | No diff. pts without t.l. necrosis & controls  Pts with t.l. necrosis impaired on verbal (P<.001) & visual memory (range, P<.001 to P = .03), language (range, P<.001 to P = .01), motor ability (P = .02), planning (P = .02), cognitive ability (P = .007), & abstract thinking (range, P = .009 to P = .04)  General intelligence & attention comparable to HC  no diff. between pts with & without TLN in mean total dosage & dosage per fr. |
| Hua  1998 | Pros | 27 | NPC | Primary  RT alone  Photon  (4680 cGy/26 fr.) | Mean= 45,14 (7.74)  (28-60) | 85 | Median 1.7 yr. post RT  (7d. – 9 yr.) | Intellectual function:  WAIS-R  Learning & Memory:  RemLET  RecLET  WSLL-R  WMS-R (Figure Memory, Logical Memory I & II, Visual Reproduction I & II.)  Language & communication:  MAE (Visual naming, Token test, Semantic Association of Verbal Fluency)  Visuospatial & visuoperceptual function:  JLO  VOSP (Silhouette, Object Decision, Progressive Silhouette)  FRT  VFD  Executive Function & manual dexterity:  WCST-M  PPB  Attention:  WMS-R (Mental Control)  PASAT-R | Matched NPC pre tx (28)  Matched HC | HC > NPC-R> NPC+R on all WAIS-R (diff. not sig.)  HC = NPC-R > NPC+R for semantic memory function  HC > NPC+R & NPC-R on most episodic memory test  HC > NPC+R for word-sequence learning  HC > NPC+R for Figure Memory subtest of WMS-R  HC > NPC+R & NPC-R visuospatial & visuo perceptual functioning  HC = NPC-R > NPC+R executive function (diff. not sig.)  HC & NPC-R > NPC+R attention Mental Control Subtest of WMS-R  No diff. in language & communication functioning |
| Gan  2011 | Pros | 10 | SCC HNC  6 Px  3 Lx  1 HPX | Primary  5 IMRT only  5 cisplatin CRT  (60-70Gy/ 20 – 35fx)  (mean 65.8 Gy / 29Gy)  (to whole brain: 0.14–4.83 Gy, mean 2.8 Gy) | Mean= 58.1 (47-66) | 80 | Mean 20 mo. Post tx  (9 – 41 mo.) | Intelligence:  WAIS-III or WASI (Vocabulary & Matrix Reasoning split half versions)  Language:  DKEFS System Verbal Fluency Test  BNT (split half version)  Memory:  HVLT–R  BVMT-R  Attention:  WAIS-III (Digit Span)  Attention/Processing speed:  TMT-A  DKEFS System Color Naming & Word Reading  Executive function:  TMT-B  DKEFS Stroop Color; Word Interference Test Inhibition & Switching  Manual Dexterity:  PPB | Normative data | 90% of pts impairment across multiple domains compared to normative data  Most severe domain was memory encoding & memory retention  normal IQ, evidence for cognitive dysfunction cognitive domains (except language) & global cognitive functioning lower than expected from IQ  For 70% of pts decline in cognitive performance across cognitive domains at least one SD  Sig. correlation between increased radiation dose to tl & worse performance on memory decoding (p=0.03)  Trend CTR greater cognitive dysfunction, but not sig. (p= 0.29) |
| Lam  2003 | Pros | 60 | NPC  40 TLI+  20 TLI- | Primary  RT only  Photon  (66- 71.2 Gy, 1.6 – 2.5 Gy/ fr.) | TLI+:  Mean= 45,7  (7.2)  TLI-:  Mean= 45.5 (5.6) | TLI+:  85  TLI-:  45 | TLI+:  Mean 5.5 yr. post RT  TLI-:  Mean 5 yr.post RT | Language:  WAIS-R (information, comprehension & similarities)  VFT  Working memory:  Digit & visual span  Verbal/Episodic memory:  RAVLT  Visual learning tests  Attention:  Monotone test for attention | Matched  HC (19)  TLI+ (40)  TLI- (20) | RT-groups lower WAIS-R scores information & comprehension subtests compared to HC  (no sig. diff. between TLI+ &TLI-)  Working memory: TLI- pts scores lower than HC  Episodic memory: pts groups worse in Rey auditory test (recall fewer items)  Pts groups worse in 20-minute delay recall & recognition scores (not sig.)  Rey visual learning: patient had lower scores across most trials  Recognition tasks: no diff.  Pts with large-volume RT plan had lower scores in WAIS-R subtests of comprehension & similarities compared to standard plan  High-dose plan did not differ on neurocognitive testing compared to standard plan  17 pts (10 TLI+, 7 TLI-) reassessed post mean 28m for progress of memory function:  TLI+ & TLI- no diff. in most verbal & visual memory tests, except TLI- recalled fewer items than TLI+ in 20-minute delayed recall  Performance digit & visual span test stable  Trend of improvement in verbal memory tests (not sig.)  Trend for worsening of visual memory (not sig.) |
| Wu  2020 | Pros | 54 | NPC | Primary  IMRT  (62.2 – 72.6 Gy ;31-33 fr. At 2.15-2.36Gy/fr) | Mean= 48.74 (12.85)  (20 – 71) | 72 | Pre RT 2 yr. post RT | Screening neurocognitive function:  MoCA | Within patient | None had cognitive impairment at BL (defined as MoCA score under 26)  19 (35.2%) showed cognitive impairments post RT  16 (29.63%) declined by 3 or more in MoCA  RT dose in pts correlated to increased cognitive impairment |
| Lee  1989 | Pros | 16 | NPC | Primary  RT only  Photon  (NPC areas: 350 cGy mean tumor dose 3x per week for 59 Gy over 6 weeks (55-63 Gy range) | Mean= 48  (9) | 69 | > 2 yr. post RT  Median 5.5 yr. (2.5 – 10.2 yr.) | Intelligence:  WAIS (information, comprehension, similarities, coding & block design)  Memory:  LMT  ALT  RFT  Global deficits: TVIP  MRT  SOA  FTT | Newly diagnosed/ pre tx NPC (21) | NPC+R < NPC-R full scale IQ, verbal IQ & performance IQ (specifically recall general information from memory, social understanding, non-verbal concept formation)  NPC+R < NPC-R delayed recall of geometric design & immediate recall of logical memory  NPC+R < NPC-R delayed verbal memory & associate learning  NPC+R < NPC-R Span of apprehension  No diff. in rest. |
| Glosser  1997 | Pros | 17 | CSB | Primary  RT only  Proton  (median 68.4 CGE (proton Gy X RBE 1.1); 66.6-72 (CGE)) | Mean= 38.6 (14.8) | 47 | Pre RT  7 mo. post RT (6-14)  25 mo. post RT (19-30)  47 mo. post RT (33-65) | Intelligence:  WAIS-R  CLAT  RPM (matched short forms)  Language & Visuospatial processing:  BNT  COWA  AN  WAIS-R (visuospatial)  Memory:  20-word & 15-design learning tests (matched form)  Attention:  Dichotic listening  Motor function:  FTT  GP  PPD | Within patient | No adverse effects in general intelligence, language, memory, & higher level attentional functions on any timepoint  Full-scale IQ scores improved (potential practice effects)  Learning for visuospatial memory test slightly improved |
| Tang  2012 | Pros | 46 | NPC | Primary  RT  12 CT  (accumulated 68-76Gy (70.2Gy median); 2 Gy/fr.) | Mean= 39.8 (15.1) | 76 | 6 yr.post RT  (1-19 yr.) | Screening neurocognitive function:  MoCA | No RI (46) | Sig. lower MoCA scores of pts with RI compared to pts without RI MoCA scores  RI+ MoCA scores= 21.32 ±2.45  RI- MoCA scores= 25.98±1.73  CT risk factor for cognitive dysfunction |
| Meyers  2000 | Pros | 19 | BST  7 SCC  5 SARC  3 ACC  2 LYM  2 or | Primary  RT  9 CT  (median 60Gy (50 – 68) 1.8 – 2 Gy/fr.) | Mean= 52.6  (2.9)  (25-69) | 37 | Mean 73.4 mo. post RT  (20 mo.-20 yr.) | Intelligence:  WAIS-R (Digit Span, arithmetic, digit symbol, similarities, block design)  Verbal memory:  VSRT  Visual memory:  BVRT  Language:  MAE  Attention/ processing speed:  TMT-A  Executive functioning:  TMT-B  WCST  Motor:  GP | Normative data | >50% difficulty learning new information  ~80% accelerated forgetting  >30% difficulties visual-motor speed, frontal lobe executive functions, & fine motor coordination  Worse scores for pts treated pre 1985 & higher dose  (Impaired performance= 1.5 SD below normative mean (6.7% of overall population expected to score in this range)  Majority normal/ close to normal intellectual function & attention  Dose associated with poorer delayed recall on verbal memory test, not with performance on any other test  No relationship with CT, total volume, tumor type or type of RT (cobalt 60 vs 6MV) |
| Shen  2016 | Pros | 106 total  28 RN-  78 RN+ | NPC | Primary  IMRT  (RN-: 70 (70-72))  21 additional CT  (RN+: 71 (66-76))  61 additional CT | RN-:  Mean= 47.9 (7.2)  RNI+=  Mean= 46.8 (8.6) | 68  73 | 5.3 yr. post RT (+/- 3.5)  6.5 yr. post RT (+/-3.9) | Screening neurocognitive function:  MoCA | Normative data  RN- | 55.1% of RN+ impaired (mean 24.8)  7.1% in RN- impaired (mean 24.8)  RN+ group performed worse than RN- group  T.l. cerebral micro bleeds as independent risk factor for cognitive dysfunction |
| Wilbers  2015 | Pros | 44 | HNC  45% Lx  14% PC  15% PA  2% NPC  17% OPC  2% HPX  6% LYM | Primary  RT  Photon  (30-70 Gy depending on tumor) | 54.3  (13) | 60 | 6.7 yr. post RT  (4.5–9.6 yr.) | Screening neurocognitive function:  MMSE  FAB  Verbal/ Episodic memory:  RAVLT  Working memory:  DST  Executive functioning:  Stroop color word-task  BSAT  Verbal fluency:  Semantic fluence task  Letter fluency task  Speed information processing:  Stroop test  SDST | Normative data | 1 patient <24 on MMSE  General worse cognitive performance in episodic memory & speed of information processing  11.4% clinically impaired in episodic memory  6.8% impaired speed of information processing  No group diff. in working memory, executive functioning & verbal fluency |
| McDowell  2019 | Pros | 102 | NPC | Primary  IMRT  94 additional CT  (101 got 70Gy/35, 1 got 66Gy) | Median=56  (32-77) | 65 | Median 7.5 yr. post RT (4.2-11.1 yr.) | Screening neurocognitive function:  MoCA | Normative data | 32% impaired MoCA scores (<23)  70 % impaired MoCa score (<26)  MoCa scores did not correlate with radiation dose, CT or TLN |
| Pruijssen  2022 | Pros | 29 | HNC  (incl. benign)  24.1% PC  27.5% PA  6.9% OPC  27.6% LYM  13.8% Ors | Primary  RT  Photon  10 concomitant CT  ( median 50 Gy, 26 – 100 Gy/2Gy fr.) | Median= 41 | 45 | Median 9.2 yr. post RT  (7.3 – 12.9 yr.) | Episodic memory:  HVLT  Working memory:  DST (backward & forward)  Executive functioning:  TMT-B/A ratio  BSAT  Stroop Color-word test  Verbal fluency:  Letter/ category fluency  Attention/Processing speed:  Stroop test  TMT-A  SDST | Normative  data | Worse episodic memory compared to normative data  17.2% impaired in episodic memory  No sig. diff. in working memory, executive functioning, verbal fluency or speed of information processing |

**Table 1.** Overview included studies, characteristics and findings;

Abbreviations: ACC= Adenoid cystic carcinoma; AN= Animal naming; ALT= Associate learning test; AVF =Action (Verb) Fluency; AVLT = Auditory verbal learning test; BST= Base of skull tumor; BL= baseline; BNT= Boston naming test; BVMT= Brief Visuospatial Memory Test; BVMT-R= Brief Visuospatial Memory Test revised; CAS= Das–Naglieri cognitive assessment system; CASB= chordomas and low-grade chondrosarcomas of skull base; CASI= Cognitive Abilities Screening Instrument; CTT= Color Trail Test Part; COWA= Controlled Oral Word Association; CR= Chemoradiation; CSB= chordomas and low-grade chondrosarcomas of skull base; CT = chemotherapy; d.= day; diff.= difference; DKEFS= Delis-Kaplan Executive Function System; DLBcnHl= Diffused large B-cell non-Hodgkin’s lymphoma; DST= Digit Span test; ES= effect size; excl. = excluding; FAB= Frontal assessment battery; fr.= fractions; FRT= Facial Recognition Test; FTT= Finger tapping test; GDS= Gordon Diagnostic System; Gy= Gray; HC= Healthy controls; HNC= head and neck cancer; HKLLT= Hong Kong List Learning Test; HP= HP; HPX= Hypopharynx; HVLT= Hopkins Verbal Learning Test; HVLT-R= Hopkins Verbal Learning Test-Revised; IMRT= Intensity-Modulated Radiation Therapy; incl.= including; JLO= Judgment of Line Orientation; LMT= logical memory test; Lx= larynx; LYM= lymphoma; MAE= Motor Agnosia and Apraxia Evaluation; Maj.= majority; max.= maximum; MRT= motor reaction time test; MMSE = Mini-Mental State Examination; MoCA= Montreal Cognitive Assessment; mo.= months; NC= Nasal cavity; n.m. = non-metastatic; NPC= nasopharyngeal carcinoma; n.r.= not reported; N SSC= squamous cell carcinoma of neck; OPC= oropharyngeal carcinoma; PA= Pleiomorphic adenoma of parotid; PDC= poorly differential carcinoma; PNS/NC = Paranasal sinus/nasal cavity; PPB= Purdue Pegboard; Pros= prospective; Px= pharynx; RAVLT total= Rey Auditory Verbal Learning Test (Total of Trials 1–5); RAVLT delayed recall= Rey Auditory Verbal Learning Test(delayed recall); RN= radiation necrosis; RFT= Rey Figure test; RPM= Raven Standard Progressive Matrices; RT= Radiotherapy; RecLET= Recent Life Events Test; SARC= sarcoma; SAVF= Semantic Association of Verbal Fluency; SD=standard deviation; SDMT= Symbol Digit Modalities Test; SDST= Symbol Digit Substitution Task; sig.= significant; SOA= Span of apprehension test; SCC= squamous cell carcinoma; tRT= tomography radiation therapy; TL = temporal lobe; TLN = temporal lobe necrosis; TLI= Temporal lobe injury; Ton SSC= squamous cell carcinoma of tonsil ; SSC= squamous cell carcinoma of tongue; TT= Token Test; TMT-A= Trail Making Test A; TMT-B= Trail Making Test B; TVIP= Tachistoscope test for visual information processing time; Tx= treatment; T&E SSC= squamous cell carcinoma of tonsil & epiglottis; UP= unknown primary; VFD= Visual Form Discrimination; VNS= Visual Naming Subtests; VOSP = Visual Object and Space Perception; WAIS-III= Wechsler Adult Intelligence Scale-III; WAIS-R= Wechsler Adult Intelligence Scale-Revised; WASI= Wechsler Abbreviated Scale of Intelligence; WCST-M= Wisconsin Card Sorting Test Modified; WSLL-R= Word Sequence Learning-Revised; WTF= Word Fluency Test; yr. = years
